# Examining the Hypertension Control Cascade in Adults With Uncontrolled Hypertension in the US

**DOI:** 10.1001/jamanetworkopen.2024.31997

**Published:** 2024-09-11

**Authors:** LaTonia C. Richardson, Adam S. Vaughan, Janet S. Wright, Fátima Coronado

**Affiliations:** 1 Division for Heart Disease and Stroke Prevention, Centers for Disease Control and Prevention, Atlanta, Georgia

## Abstract

**Importance:**

Uncontrolled hypertension is a major contributor to cardiovascular disease (CVD) in the US.

**Objective:**

To determine the prevalence of hypertension control cascade outcomes (hypertension awareness, treatment recommendations, and medication use) among individuals with uncontrolled hypertension to inform action across cascade levels.

**Design, Setting, and Participants:**

This weighted cross-sectional study used January 2017 to March 2020 National Health and Nutrition Examination Survey (NHANES) data from noninstitutionalized adults aged 18 years or older in the US with uncontrolled hypertension. Data analysis occurred from January to February 2024.

**Exposure:**

Calendar year of response to the NHANES survey.

**Main Outcomes and Measures:**

Mean blood pressure (BP) was computed using up to 3 measurements. Uncontrolled hypertension was defined as systolic BP of 130 mm Hg or greater or diastolic BP of 80 mm Hg or greater, regardless of medication use. Outcomes included patient awareness of hypertension, treatment recommendations, and medication use. To estimate population totals by subgroup, the age-standardized proportion of each outcome was multiplied by the estimated number of adults with uncontrolled hypertension.

**Results:**

The study included 3129 US adults with uncontrolled hypertension (1675 male [weighted percentage, 52.3%]; 775 aged 18 to 44 years [weighted percentage, 29.4%]; 1306 aged 45 to 64 years [weighted percentage, 41.4%]; 1048 aged 65 years or older [weighted percentage, 29.2%]), resulting in a population estimate of 100.4 million adults (weighted percentage, 83.7%) with uncontrolled hypertension. More than one-half of study participants (57.8 million adults [weighted percentage, 57.6%]) were unaware that they had hypertension, and of the 35.0 million who were aware and met criteria for antihypertensive medication, 24.8 million (weighted percentage, 70.8%) took the medication but had hypertension that remained uncontrolled. These negative outcomes in the hypertension control cascade occurred across demographic groups, with notably high prevalence among younger adults and individuals engaged in health care. Among an estimated 30.1 million adults aged 18 to 44 years with hypertension, 10.4 of 11.3 million females (weighted percentage, 91.8%) and 17.7 million of 18.8 million males (weighted percentage, 94.3%) had uncontrolled hypertension. Of the 10.4 million females, 7.2 million (weighted percentage, 68.8%) were unaware of their hypertension status, and of the 17.7 million males, 12.0 million (weighted percentage, 68.1%) were unaware. Additionally, 9.9 of 13.0 million adults with uncontrolled hypertension (weighted percentage, 75.7%) reported no health care visits in the past year and were unaware. Conversely, among 70.6 million adults with uncontrolled hypertension reporting 2 or more health care visits, approximately one-half (36.6 million [weighted percentage, 51.8%]) were unaware.

**Conclusions and Relevance:**

In this cross-sectional study, more than 50% of adults with uncontrolled hypertension in the US were unaware of their hypertension and were untreated, and 70.8% of those who were treated had hypertension that remained uncontrolled. These findings have serious implications for the nation’s overall health given the association of hypertension with increased risk for CVD.

## Key Points

**Question:**

What are the hypertension control cascade estimates among adults with uncontrolled hypertension in the US?

**Findings:**

This cross-sectional study of 3129 adults aged 18 years or older with uncontrolled hypertension found that uncontrolled hypertension prevalence overall was high at 83.7%. Younger adults aged 18 to 44 years with hypertension had especially high prevalence of uncontrolled hypertension of which they were unaware, with marked differences by health care utilization.

**Meaning:**

The findings of this study suggest there are opportunities to increase hypertension awareness and treatment to reduce cardiovascular disease and improve the nation’s overall health.

## Teaser

This cross-sectional study provides estimates of hypertension awareness, treatment recommendations, and medication use among individuals with uncontrolled hypertension in the US.

## Introduction

Approximately 120 million adults in the US (48.1%) have hypertension; of those, 92.9 million (77.4%) have uncontrolled hypertension,^1^ with disparities in hypertension prevalence and control by sex,^2,3,4^ age group,^5,6^ and race and ethnicity.^7,8,9,10^ Uncontrolled hypertension, which costs the nation $131 to $198 billion yearly,^11^ is a leading factor associated with increased risk of cardiovascular disease (CVD) mortality and events, including heart attack and stroke, and is also associated with an increased risk of diabetes, chronic kidney disease (CKD), and cognitive decline.^12,13^

The hypertension control cascade is a nested framework for understanding and intervening on hypertension at different levels including awareness, treatment, and control.^1,14,15,16^ Individuals must first be aware of their diagnosis to be eligible for recommended treatments and must then be treated to achieve control. Prior studies have examined the hypertension cascade by applying the previous Joint National Committee (JNC) blood pressure (BP) guidelines^17^ to the total US population singly stratified by various sociodemographic variables.^9,15,18^ However, assessing the cascade among all adults in the US, including those with controlled hypertension, obscures variation by control status. Therefore, limiting cascade outcome measures to individuals with uncontrolled hypertension can inform at which cascade level evidence-based strategies, programs, and interventions may be most useful among this at-risk population. Additionally, presenting results by sociodemographic groups and by subgroups within sex can help to tailor solutions and inform efforts to reduce disparities.

In 2017, the American College of Cardiology/American Heart Association (ACC/AHA) updated hypertension guidelines for adults aged 18 years or older, defining hypertension as systolic BP (SBP)greater than or equal to 130 mm Hg and diastolic BP (DBP) greater than or equal to 80 mm Hg. This definition expanded eligibility for pharmacologic treatment and lifestyle modification for BP management, replacing the prior JNC guidelines.^16,19^ Therefore, this study uses current hypertension guidelines to present the hypertension control cascade (awareness, treatment eligibility, and medication use) from January 2017 to March 2020 among adults aged 18 years or older in the US with uncontrolled hypertension, stratified by demographic and socioeconomic factors.

## Methods

### Data Source

This cross-sectional study was approved by the Centers for Disease Control and Prevention (CDC) and followed the Strengthening the Reporting of Observational Studies in Epidemiology (STROBE) reporting guideline. We used the January 2017 to March 2020 National Health and Nutrition Examination Survey (NHANES), a nationally representative, cross-sectional survey of the US civilian, noninstitutionalized population. NHANES methodology, including the process for obtaining written informed consent from all study participants, has been described elsewhere.^20^ NHANES data are typically published as 2-year survey cycles. Data for the 2019 to 2020 survey cycle, which stopped collection in March 2020 due to the COVID-19 pandemic and therefore excludes pandemic-related impacts, were combined with 2017 to 2018 data to achieve a nationally representative sample and released as a public use dataset.^21^ We used this combined dataset.

Overall, 8965 persons aged 18 years or older completed the NHANES examination during January 2017 to March 2020. We excluded participants who reported pregnancy during the survey (87 participants), had missing BP measurements (930 participants), had missing current BP medication use (3 participants), or had unknown values for other covariates (617 participants).

NHANES data are publicly available. This secondary analysis was reviewed by CDC and was conducted in adherence with applicable federal law and CDC policy.

### Hypertension Definition

SBP and DBP were calculated as the mean of up to 3 consecutive BP measurements. We defined hypertension as having a BP reading meeting the 2017 ACC/AHA guidelines definition (SBP ≥130 mm Hg or DBP ≥80 mm Hg) or self-reported current use of BP-lowering medication (regardless of BP reading). We defined uncontrolled hypertension consistent with the 2017 ACC/AHA guidelines, with or without current use of BP-lowering medication.

### Hypertension Awareness Definition

Participants were asked the question, “Have you ever been told by a doctor or health professional that you had hypertension, also called high blood pressure?” Those who responded yes were considered aware of their hypertension status.

### Treatment Recommendation Definitions

Based on the 2017 ACC/AHA guidelines, participants who were aware of their hypertension status were considered as meeting criteria for lifestyle modifications and pharmacologic treatment if they reported current BP medication use, had stage 2 hypertension (SBP ≥140 mm Hg or DBP ≥90 mm Hg), had stage 1 hypertension (SBP, 130-139 mm Hg; DBP, 80-89 mm Hg) and an existing or high risk of developing CVD (atherosclerotic CVD [ASCVD] score ≥10%), or were aged 65 years or older.^16^ Meeting criteria for lifestyle modifications alone was defined as having stage 1 hypertension with a low risk of developing CVD (ASCVD score <10%). Participants unaware of their hypertension status were considered to not meet criteria for any recommendations.

### Medication Use Definition

Among participants meeting criteria for lifestyle modifications and pharmacologic treatment, we defined participants as currently taking BP-lowering medication. This was determined using self-reported status.

### Population Characteristics

Age was categorized as 18 to 44 years, 45 to 64 years, and 65 years or older. Self-reported race and ethnicity were queried in the same survey question and categorized as Hispanic (Mexican American and other Hispanic combined), non-Hispanic Asian, non-Hispanic Black, non-Hispanic White, and non-Hispanic other (includes multiracial individuals and any other non-Hispanic group other than non-Hispanic Asian, non-Hispanic Black, and non-Hispanic White). We further analyzed age and race and ethnicity within 2 sex categories: male and female. Race and ethnicity were assessed due to racial disparities in hypertension prevalence and control.^7,8,9,10^

Self-reported educational attainment was categorized as less than high school, high school graduate or equivalent, some college or associate’s degree, and college graduate or above. Federal income-to-poverty ratio was defined as the participant’s family income divided by the federal poverty level and categorized as less than 1.30%, 1.30% to 3.50%, and greater than 3.50%.^22^ Participants reporting having Medicare, private, or other public health insurance were considered to have health insurance. We determined the number of health care visits during the past year based on the question, “During the last 12 months how many times have you seen a doctor or other health professional about your health at a doctor’s office, a clinic, hospital emergency department, at home or some other place? Do not include times you were hospitalized overnight.” Responses were categorized as 0 visits, 1 visit, and 2 or more visits.

We further analyzed cooccurring health conditions. Participants were categorized based on body mass index (BMI; calculated as weight in kilograms divided by height in meters squared) as normal or underweight (<25.0), overweight (25.0-29.9), and obese (≥30.0); BMI was predetermined in the downloaded NHANES dataset. Participants were considered to have diabetes based on self-report, having a hemoglobin A1c value of 6.5% or greater (to convert to proportion of total hemoglobin, multiply by 0.01), or having a fasting plasma glucose level of 126 mg/dL or greater (to convert to millimoles per liter, multiply by 0.0555). We defined participants as having CKD based on an estimated glomerular filtration rate less than 60 mL/min/1.73 m^2^ or a urine albumin-to-creatinine ratio of 30 mg/g or greater. Participants were considered to have a history of clinical CVD based on self-reported diagnosis of coronary heart disease, congestive heart failure, acute myocardial infarction, angina, or stroke.

### Statistical Analysis

We determined unweighted counts, age-standardized weighted counts, age-standardized weighted prevalence and corresponding 95% CIs for each participant characteristic. Values were age-standardized to the 2000 standard US population.^23,24^

To estimate population totals by subgroup, we multiplied the age-standardized proportion of each outcome by the estimated number of adults with uncontrolled hypertension, which was calculated based on the National Center for Health Statistics (NCHS) civilian noninstitutionalized population totals for adults aged 18 years or older from January 2017 to March 2020^25^ and NHANES estimated proportions of adults with hypertension and uncontrolled hypertension. Using the stepped approach of the hypertension control cascade, we calculated weighted prevalence and 95% CIs for (1) uncontrolled hypertension (among all with hypertension), (2) hypertension awareness (among all with uncontrolled hypertension), (3) meeting criteria for treatment recommendations (among those aware of their hypertension status), and (4) antihypertensive medication use (among those aware and meeting criteria for lifestyle modifications plus medication).

We calculated prevalence estimates overall and by age, sex, race and ethnicity, age within sex, race and ethnicity within sex, and sociodemographic variables. Prevalence data and population estimates were suppressed in accordance with NCHS standards for presenting proportions.^26^

All analyses used sampling weights^16^ (SAS version9.4 [SAS Institute]) to account for NHANES multistage, clustered sample design. We used R software version 4.0.5 (R Foundation for Statistical Computing) for visualizations. Statistical significance was considered a 2-sided *P* < .05. Data analysis was conducted from January to February 2024.

## Results

### Population Characteristics

After applying exclusionary criteria to those who had completed the NHANES examination, there were 7328 individuals, of which 3954 (54.0%) had hypertension, and 3129 of those with hypertension (79.1%) were uncontrolled. These 3129 adults with uncontrolled hypertension constituted our study sample and had the following characteristics: 1675 male (weighted percentage, 52.3%), 775 aged 18 to 44 years (weighted percentage, 29.4%), 1306 aged 45 to 64 years (weighted percentage, 41.4%), 1048 aged 65 years or older (weighted percentage 29.2%), 589 Hispanic (weighted percentage 13.4%), 324 non-Hispanic Asian (weighted percentage, 5.3%), 973 non-Hispanic Black (weighted percentage 13.7%]), 148 non-Hispanic other (weighted percentage, 4.5%), 1095 non-Hispanic White (weighted percentage 63.2%) (Table 1). Most study participants were privately insured (1569 participants [weighted percentage, 60.3%]) and saw a health care clinician 2 or more times in the past year (2181 participants [weighted percentage, 69.2%]). Furthermore, approximately one-half of participants had obesity (1505 participants [weighted percentage, 50.3%]), 1 in 5 participants had a history of diabetes (806 participants [weighted percentage, 20.7%]) or CKD (806 participants [weighted percentage, 20.7%]), and 561 (weighted percentage, 16.0%) had a history of ASCVD. After population estimation, there were an estimated 120 million individuals with hypertension among whom 100.4 million were uncontrolled (weighted percentage 83.7%).

**Table 1.** Characteristics of US Adults Aged 18 Years or Older With Uncontrolled Hypertension, January 2017 to March 2020

Characteristic	Participants, No. (weighted %) [95% CI] (N =3129)
Sex	
Male	1675 (52.3) [48.2-56.3]
Female	1454 (47.7) [43.7-51.8]
Age group, y	
18-44	775 (29.4) [25.5-33.3]
45-64	1306 (41.4) [39.1-43.7]
≥65	1048 (29.2) [25.4-33.0]
Race and ethnicity	
Hispanic	589 (13.4) [10.6-16.1]
Non-Hispanic Asian	324 (5.3) [3.7-6.8]
Non-Hispanic Black	973 (13.7) [9.9-17.4]
Non-Hispanic White	1095 (63.2) [57.1-69.2]
Non-Hispanic other^a^	148 (4.5) [3.4-5.7]
Males by demographic	
Age	
18-44 y, No./Total No. (weighted %) [95% CI]	469/1675 (36.3) [31.9-40.8]
45-64 y, No./Total No. (weighted %) [95% CI]	679/1675 (39.3) [36.2-42.3]
≥65 y, No./Total No. (weighted %) [95% CI]	527/1675 (24.4) [20.5-28.3]
Race and ethnicity	
Hispanic, No./Total No. (weighted %) [95% CI]	326/1675 (15.3) [11.9-18.8]
Non-Hispanic Asian, No./Total No. (weighted %) [95% CI]	171/1675 (5.4) [3.8-7.0]
Non-Hispanic Black, No./Total No. (weighted %) [95% CI]	497/1675 (12.3) [8.7-15.9]
Non-Hispanic White, No./Total No. (weighted %) [95% CI]	588/1675 (61.6) [54.2-69.1]
Non-Hispanic other, No./Total No. (weighted %) [95% CI]^a^	93/1675 (5.4) [3.6-7.1]
Females by demographic	
Age	
18-44 y, No./Total No. (weighted %) [95% CI]	306/1454 (21.8) [18.0-25.5]
45-64 y, No./Total No. (weighted %) [95% CI]	627/1454 (43.8) [40.6-47.0]
≥65 y, No./Total No. (weighted %) [95% CI]	521/1454 (34.4) [29.0-39.0]
Hispanic, No./Total No. (weighted %) [95% CI]	263/1454 (11.2) [8.2-14.2]
Race and ethnicity	
Non-Hispanic Asian, No./Total No. (weighted %) [95% CI]	153/1454 (5.2) [3.4-7.0]
Non-Hispanic Black, No./Total No. (weighted %) [95% CI]	476/1454 (15.1) [10.7-19.6]
Non-Hispanic White, No./Total No. (weighted %) [95% CI]	507/1454 (64.8) [58.4-71.2]
Non-Hispanic other, No./Total No. (weighted %) [95% CI]^a^	Suppressed^b^
Education	
Less than high school	612 (12.6) [10.7-14.4]
High school graduate, general educational development, or equivalent	784 (29.2) [25.9-32.5]
Some college or associates degree	1027 (32.0) [28.7-35.2]
College graduate or above	681 (25.8) [21.4-30.1]
Federal income to poverty ratio, %	
≤130	765 (16.2) [13.9-18.4]
131-350	1099 (33.5) [29.9-37.2]
>350	859 (39.5) [35.6-43.4]
Health insurance status	
Private	1569 (60.3) [56.8-63.8]
Medicare	619 (13.8) [11.5-16.1]
Other public	504 (13.8) [11.7-16.0]
Uninsured	421 (11.7) [9.1-14.3]
Health care visits in the past year, No.	
0	430 (14.1) [11.2-17.0]
1	511 (16.6) [14.4-18.8]
≥2	2181 (69.2) [66.1-72.2]
Body mass index categories^c^	
Normal or underweight (<25.0)	611 (18.2) [15.9-20.5]
Overweight (25.0-29.9)	958 (30.5) [28.0-32.9]
Obese (≥30.0)	1505 (50.3) [47.0-53.6]
Other chronic health conditions	
Diabetes	806 (20.7) [18.8-22.6]
Chronic kidney disease	806 (20.7) [18.8-22.6]
History of atherosclerotic cardiovascular disease	561 (16.0) [13.1-18.9]

^a^ Includes those who self-reported being multiracial or a non-Hispanic race other than Asian, Black, or White.

^b^ Estimate suppressed in accordance with National Center for Health Statistics standards for presenting proportions.^26^

^c^ Body mass index is calculated as weight in kilograms divided by height in meters squared; body mass index was predetermined in the downloaded National Health and Nutrition Examination Survey dataset.

### Hypertension Control Cascade Among Adults in the US With Uncontrolled Hypertension

**Figure 1.**
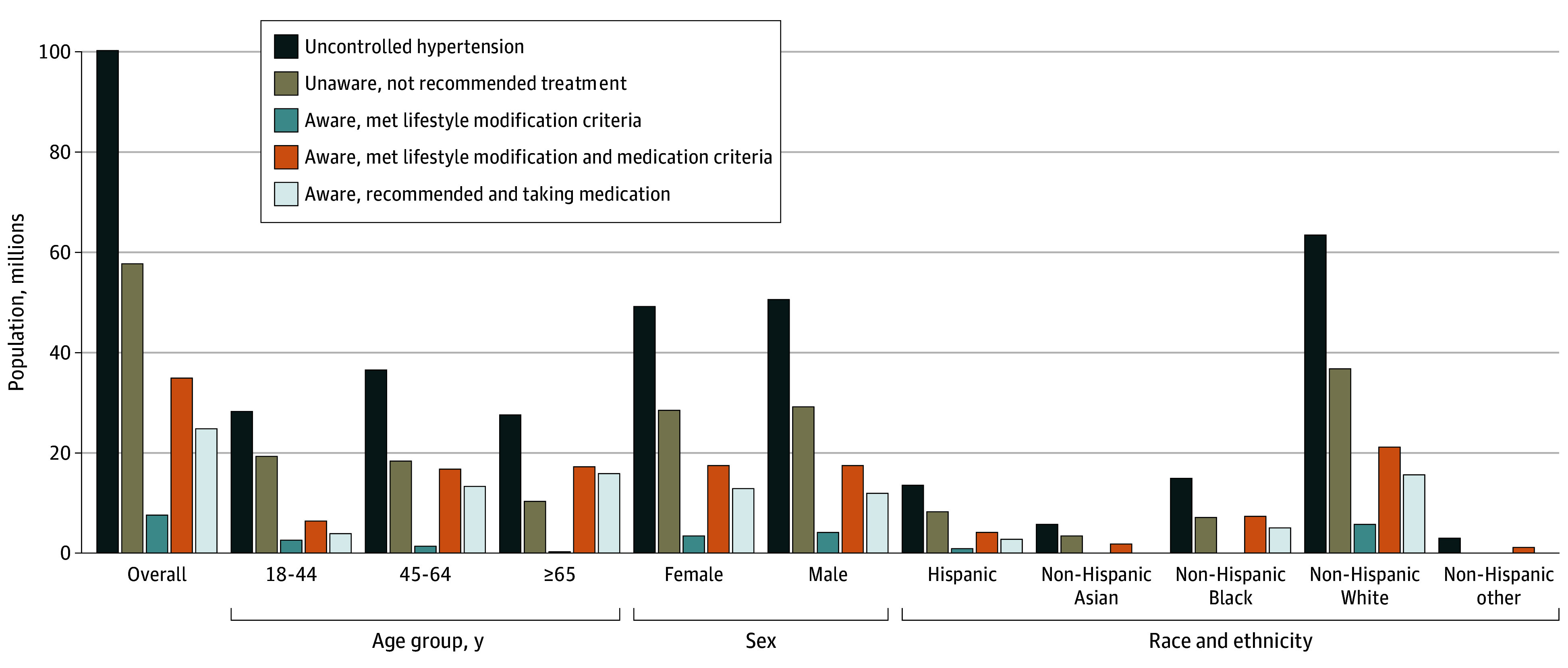
Hypertension Control Cascade Population Estimates Among Adults Aged 18 Years or Older in the US With Uncontrolled Hypertension, Overall and by Age, Sex, and Race and Ethnicity, January 2017 to March 2020 The population meeting lifestyle modification criteria and meeting lifestyle modifications and medication criteria were calculated among individuals who were aware of their hypertension status and were independent of medication use. The population taking blood pressure medication was calculated among those meeting lifestyle modifications and medication criteria. Adults aged 65 years or older with hypertension are not eligible for only lifestyle modification. Missing bars reflect estimates suppressed in accordance with National Center for Health Statistics Standards for presenting proportions^26^. Non-Hispanic other included those who self-reported multiracial or any non-Hispanic ethnicity other than Asian, Black, or White.

Among adults in the US aged 18 years or older with hypertension from January 2017 to March 2020, the age-standardized prevalence of uncontrolled hypertension was 83.7% (95% CI, 80.6%-86.8%) (Table 2 and Figure 1). Overall, an estimated 57.8 million adults (weighted percentage, 57.6%) with uncontrolled hypertension were unaware, while 7.6 million (weighted percentage, 17.8%) were aware and met lifestyle modification criteria. Among 35.0 million adults with uncontrolled hypertension who met criteria for medication from January 2017 to March 2020, 24.8 million (weighted percentage, 70.8%) reported taking medication.

**Table 2.** Age-Standardized Hypertension Cascade Prevalence Estimates Among Adults Aged 18 Years or Older in the US With Hypertension, Overall and by Age, Race And Ethnicity, and Sex, January 2017 to March 2020

Characteristic by demographic	Overall		Males		Females	
	Sample size (estimated population in millions)	Weighted % (95% CI)	Sample size (estimated population in millions)	Weighted % (95% CI)	Sample size (estimated population in millions)	Weighted % (95% CI)
Overall						
Uncontrolled hypertension^a^	3129 (100.4)	83.7 (80.6-86.8)	1675 (50.7)	83.4 (79.4-87.3)	1454 (49.3)	83.6 (78.8-88.4)
Unaware, not recommended treatment^b^	1442 (57.8)	57.6 (53.3-61.8)	834 (29.3)	57.7 (50.8-64.5)	608 (28.4)	57.6 (51.4-63.8)
Aware, met criteria for lifestyle modifications^c^	89 (7.6)	17.8 (11.7-23.9)	55 (4.0)	18.7 (10.1-27.2)	34 (3.4)	16.4 (7.7-25.2)
Aware, met criteria for lifestyle modifications plus medication^c^	1598 (35.0)	82.2 (76.1-88.3)	786 (17.5)	81.3 (72.8-89.9)	812 (17.5)	83.6 (74.8-92.3)
Aware, currently taking BP medication^d^	1299 (24.8)	70.8 (63.5-78.2)	618 (11.9)	68.4 (57.7-79.0)	681 (12.9)	73.7 (63.6-83.8)
Ages 18-44 y						
Uncontrolled hypertension^a^	775 (28.3)	93.4 (90.3-96.4)	469 (17.7)	94.3 (90.8-97.7)	306 (10.4)	91.8 (87.2-96.4)
Unaware, not recommended treatment^b^	520 (19.4)	68.4 (64.3-72.4)	316 (12.0)	68.1 (62.3-73.9)	204 (7.2)	68.8 (62.8-74.8)
Aware, met criteria for lifestyle modifications^c^	59 (2.6)	29.2 (20.2-38.1)	40 (1.7)	30.2 (17.8-42.7)	19 (0.9)	27.2 (13.1-41.2)
Aware, met criteria for lifestyle modifications plus medication^c^	196 (6.4)	70.8 (61.9-79.8)	113 (3.9)	69.8 (57.3-82.2)	83 (2.4)	72.8 (58.8-86.9)
Aware, currently taking BP medication^d^	108 (3.8)	59.9 (50.1-69.6)	59 (2.3)	58.4 (45.2-71.7)	49 (1.5)	62.4 (47.9-77.0)
Ages 45-64 y						
Uncontrolled hypertension^a^	1306 (36.5)	74.5 (71.3-77.6)	679 (17.9)	73.2 (68.6-77.7)	627 (18.5)	75.8 (70.5-81.1)
Unaware, not recommended treatment^b^	570 (18.3)	50.2 (45.2-55.2)	322 (9.0)	50.4 (41.4-59.4)	248 (9.3)	50.0 (42.9-57.1)
Aware, met criteria for lifestyle modifications^c^	30 (1.4)	7.7 (3.4-12.1)	Suppressed^e^	Suppressed^e^	15 (0.6)	6.7 (2.4-11.1)
Aware, met criteria for lifestyle modifications plus medication^c^	706 (16.7)	92.3 (87.9-96.6)	342 (8.1)	91.2 (84.7-97.8)	364 (8.6)	93.3 (88.9-97.6)
Aware, currently taking BP medication^d^	553 (13.2)	78.8 (73.5-84.0)	261 (6.1)	74.7 (65.8-83.6)	292 (7.1)	82.6 (76.9-88.4)
Ages ≥65 y						
Uncontrolled hypertension^a^	1048 (27.5)	69.7 (66.7-72.7)	527 (11.6)	67.2 (62.9-71.5)	521 (15.9)	71.7 (67.1-76.4)
Unaware, not recommended treatment^b^	352 (10.2)	37.0 (33.3-40.6)	196 (4.4)	38.1 (31.8-44.3)	156 (5.8)	36.1 (30.8-41.4)
Aware, met criteria for lifestyle modifications^c^	0 (0.0)	0 (0.0)	0 (0.0)	0 (0.0)	0 (0.0)	0 (0.0)
Aware, met criteria for lifestyle modifications plus medication^c^	696 (17.3)	100.0 (100.0-100.0)	331 (7.2)	100.0 (100.0-100.0)	365 (10.2)	100.0 (100.0-100.0)
Aware, currently taking BP medication^d^	638 (15.8)	91.1 (87.7-94.4)	298 (6.3)	88.2 (82.7-93.7)	340 (9.5)	93.2 (89.5-96.9)
Hispanic						
Uncontrolled hypertension^a^	589 (13.5)	86.1 (80.9-91.3)	326 (7.7)	84.9 (78.3-91.2)	Suppressed^e^	Suppressed^e^
Unaware, not recommended treatment^b^	305 (8.3)	61.8 (54.4-69.1)	189 (4.8)	63.1 (53.4-72.8)	Suppressed^e^	Suppressed^e^
Aware, met criteria for lifestyle modifications^c^	13 (0.9)	18.3 (10.2-26.5)	11 (0.6)	22.5 (13.6-31.9)	Suppressed^e^	Suppressed^e^
Aware, met criteria for lifestyle modifications plus medication^c^	271 (4.2)	81.7 (73.5-89.8)	126 (2.2)	77.5 (68.1-86.4)	Suppressed^e^	Suppressed^e^
Aware, currently taking BP medication^d^	213 (2.7)	65.0 (51.1-78.9)	Suppressed^e^	Suppressed^e^	Suppressed^e^	Suppressed^e^
Non-Hispanic Asian						
Uncontrolled hypertension^a^	324 (5.7)	85.7 (76.6-93.3)	171 (2.9)	84.0 (72.3-93.6)	153 (2.7)	87.4 (77.8-95.4)
Unaware, not recommended treatment^b^	172 (3.4)	60.5 (49.5-71.5)	Suppressed^e^	Suppressed^e^	Suppressed^e^	Suppressed^e^
Aware, met criteria for lifestyle modifications^c^	Suppressed^e^	Suppressed^e^	Suppressed^e^	Suppressed^e^	Suppressed^e^	Suppressed^e^
Aware, met criteria for lifestyle modifications plus medication^c^	141 (1.9)	83.3 (69.3-97.3)	Suppressed^e^	Suppressed^e^	Suppressed^e^	Suppressed^e^
Aware, currently taking BP medication^d^	Suppressed^e^	Suppressed^e^	Suppressed^e^	Suppressed^e^	Suppressed^e^	Suppressed^e^
Non-Hispanic Black						
Uncontrolled hypertension^a^	973 (14.9)	87.6 (84.2-91.1)	497 (6.8)	90.5 (86.1-94.7)	476 (8.1)	85.2 (80.4-90.0)
Unaware, not recommended treatment^b^	360 (7.1)	47.4 (40.7-54.2)	202 (3.4)	49.6 (39.4-59.7)	158 (3.7)	45.8 (37.4-54.3)
Aware, met criteria for lifestyle modifications^c^	Suppressed^e^	Suppressed^e^	Suppressed^e^	Suppressed^e^	Suppressed^e^	Suppressed^e^
Aware, met criteria for lifestyle modifications plus medication^c^	596 (7.4)	94.2 (89.8-98.5)	286 (3.3)	95.0 (90.4-99.4)	310 (4.1)	93.2 (87.2-99.2)
Aware, currently taking BP medication^d^	472 (5.0)	68.3 (58.5-78.2)	222 (2.1)	64.8 (50.6-78.9)	250 (3.0)	72.3 (58.9-85.6)
Non-Hispanic White						
Uncontrolled hypertension^a^	1095 (63.4)	82.3 (77.5-87.0)	588 (31.7)	81.4 (75.8-87.0)	507 (31.7)	83.0 (75.2-90.8)
Unaware, not recommended treatment^b^	531 (36.7)	57.8 (51.6-64.0)	295 (17.9)	56.4 (46.7-66.1)	236 (18.9)	59.8 (50.1-69.4)
Aware, met criteria for lifestyle modifications^c^	41 (5.7)	21.3 (12.1-30.5)	23 (2.9)	20.8 (8.5-33.1)	18 (2.9)	22.5 (8.9-36.0)
Aware, met criteria for lifestyle modifications plus medication^c^	523 (21.1)	78.7 (69.5-87.9)	270 (10.9)	79.2 (66.9-91.5)	253 (9.9)	77.5 (64.0-91.1)
Aware, currently taking BP medication^d^	445 (15.6)	74.0 (61.5-86.5)	Suppressed^e^	Suppressed^e^	Suppressed^e^	Suppressed^e^
Non-Hispanic other^f^						
Uncontrolled hypertension^a^	148 (3.0)	83.9 (72.8-94.6)	Suppressed^e^	Suppressed^e^	Suppressed^e^	Suppressed^e^
Unaware, not recommended treatment^b^	Suppressed^e^	Suppressed^e^	Suppressed^e^	Suppressed^e^	Suppressed^e^	Suppressed^e^
Aware, met criteria for lifestyle modifications^c^	Suppressed^e^	Suppressed^e^	Suppressed^e^	Suppressed^e^	Suppressed^e^	Suppressed^e^
Aware, met criteria for lifestyle modifications plus medication^c^	67 (1.0)	80.6 (65.6-94.9)	Suppressed^e^	Suppressed^e^	26 (0.5)	84.5 (72.9-96.1)
Aware, currently taking BP medication^d^	Suppressed^e^	Suppressed^e^	Suppressed^e^	Suppressed^e^	Suppressed^e^	Suppressed^e^

Abbreviation: BP, blood pressure.

^a^ Among all adults in the US with hypertension.

^b^ Among adults in the US with uncontrolled hypertension; individuals who were unaware of their hypertension status were considered to not have been recommended treatment.

^c^ Among adults in the US with uncontrolled hypertension who were aware of their hypertension status.

^d^ Among adults in the US with uncontrolled hypertension who were aware of their hypertension status and met the 2017 criteria for lifestyle modifications plus medication.

^e^ Estimate suppressed in accordance with National Center for Health Statistics standards for presenting proportions.^26^

^f^ Includes those who self-reported being multiracial or a non-Hispanic race other than Asian, Black, or White.

Across age groups, the prevalence of uncontrolled hypertension was high, ranging from 69.7% (95% CI, 66.7%-72.7%) among adults aged 65 years or older to 93.4% (95% CI, 90.3%-96.4%) among adults aged 18 to 44 years. Unawareness was high among adults aged 18 to 44 years (19.4 million individuals [weighted percentage, 68.4%]). Among 17.3 million adults aged 65 years or older with uncontrolled hypertension who met criteria for medication, nearly all (15.8 million individuals [weighted percentage, 91.1%]) took medication.

Across racial and ethnic groups, measures of the hypertension control cascade remained high, with a high age-standardized prevalence of uncontrolled hypertension for most groups (Table 2 and Figure 1). Nearly two-thirds of non-Hispanic Asian adults (3.4 of 5.7 million [weighted percentage, 60.5%) were unaware that they had hypertension, compared with less than one-half of non-Hispanic Black adults (7.1 of 14.9 million [weighted percentage, 47.4%]) and more than one-half of non-Hispanic White adults (36.7 million of 63.4 million [weighted percentage, 57.8%]). Across racial and ethnic groups, most adults with uncontrolled hypertension who met criteria for medication reported taking antihypertensive medication.

**Figure 2.**
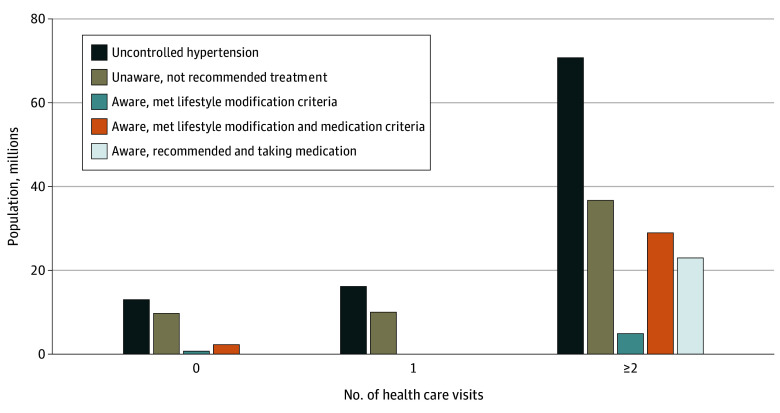
Hypertension Control Cascade Population Estimates Among Adults Aged 18 Years or Older in the US With Uncontrolled Hypertension by Number of Health Care Visits in the Past Year, January 2017 to March 2020 The population meeting lifestyle modification criteria and meeting lifestyle modifications and medication criteria were calculated among individuals who were aware of their hypertension status and were independent of medication use. The population taking blood pressure medication was calculated among those meeting lifestyle modifications and medication criteria. Missing bars reflect estimates suppressed in accordance with National Center for Health Statistics Standards for presenting proportions.^26^

The prevalence of uncontrolled hypertension and other measures of the hypertension control cascade remained high across subgroups defined by BMI status, educational attainment, income level, and insurance status (eTable and eFigure 1 in Supplement 1). Notably, 9.9 of 13.0 million adults with uncontrolled hypertension (weighted percentage, 75.7%) reported no health care visits in the past year between January 2017 and March 2020 and were unaware (eTable in Supplement 1 and Figure 2). Conversely, approximately one-half of adults with uncontrolled hypertension reporting 2 or more health care visits in the past year were unaware (36.6 of 70.6 million adults [weighted percentage, 51.8%]). Of the 29.0 million who were aware and met criteria for BP medication, 23.0 million (weighted percentage, 79.4%) reported taking medication to control hypertension, despite hypertension remaining uncontrolled.

### Hypertension Control Cascade in Adults in the US With Uncontrolled Hypertension, Stratified by Sex

When stratified by sex, hypertension control cascade measures generally were high across age groups and race and ethnicity groups (Table 2 and eFigure 2 in Supplement 1). The age-standardized prevalence of uncontrolled hypertension was 94.3% (95% CI, 90.8%-97.7%) among males aged 18 to 44 years, 73.2% (95% CI, 68.6%-77.7%) among males aged 45 to 64 years, and 67.2% (95% CI, 62.9%-71.5%) among males aged 65 years or older. More than two-thirds of males aged 18 to 44 years (12.0 of 17.7 million males [weighted percentage, 68.1%]) were unaware of their hypertension status. Although more than two-thirds of males aged 18 to 44 years who were aware of their uncontrolled hypertension status met the criteria for antihypertension medication (3.9 of 5.6 million males [weighted percentage, 69.8%]), more than one-half (2.3 million males [weighted percentage, 58.4%]) reported currently taking medication. For each race and ethnicity group with reportable data, the age-standardized prevalence of uncontrolled hypertension was more than 80.0%. Nearly all non-Hispanic Black males who were aware of their uncontrolled hypertension status met criteria for BP medication (3.3 of 3.5 million males [weighted percentage, 95.0%]), but only about two-thirds of those meeting the criteria (2.1 million of 3.3 million males [weighted percentage, 64.8%]) reported currently taking medication.

Of an estimated 11.3 million females aged 18 to 44 years with hypertension, 10.4 million (weighted percentage, 91.8%) were uncontrolled. Although more than two-thirds of females aged 18 to 44 years with uncontrolled hypertension (7.2 million females [weighted percentage, 68.8%]) and one-half of females aged 45 to 64 years (9.3 of 18.5 million females [weighted percentage, 50.0%]) with uncontrolled hypertension were unaware of their hypertension status, less than one-half of females aged 65 years or older were unaware (5.8 of 15.9 million females [weighted percentage, 36.1%]). Furthermore, although more than 80% of females aged 45 to 64 years who met the criteria for medication reported taking medication (7.1 of 8.6 million females [weighted percentage, 82.6%]) and more than 90% of females aged 65 years or older reported taking BP medication (9.5 million of 10.2 million females who were aware and met criteria for medication [weighted percentage, 93.2%]) approximately two-thirds of females aged 18 to 44 years (1.5 million of 2.4 million females who were aware and met criteria for medication [weighted percentage, 62.4%]) reported taking medication.

For each race and ethnicity group with reportable data, more than 80% of females had uncontrolled hypertension (eFigure 2 in Supplement 1). Nearly one-half of non-Hispanic Black females with uncontrolled hypertension were unaware of their status (3.7 of 8.1 million females [weighted percentage, 45.8%]), and although 4.1 million non-Hispanic Black females (weighted percentage, 93.2%) were aware of their status and met the criteria for BP medication, only 3.0 million (weighted percentage, 72.3%) reported taking medication.

## Discussion

In this nationally representative cross-sectional study, we examined the hypertension control cascade among adults in the US with uncontrolled hypertension. From January 2017 to March 2020, more than three-quarters (100.4 of 120 million [weighted percentage, 83.7%]) of adults in the US aged 18 years or older with hypertension had uncontrolled hypertension, with approximately one-half (57.8 of 100.4 million, [weighted percentage, 57.6) being unaware of their condition (and therefore remaining untreated). Of the 35.0 million individuals with uncontrolled hypertension meeting criteria for antihypertensive medication, more than two-thirds (24.8 million individuals) reported taking medication but remained uncontrolled. These negative outcomes occurred across sociodemographic groups. Notably, we identified high unawareness and lack of control among younger adults aged 18 to 44 years, including both males and females, and marked differences across the measures of the cascade by health care utilization. Our findings emphasize the pressing need for implementing evidence-based strategies to improve hypertension awareness and management among adults with uncontrolled hypertension in the US, including among females of reproductive age, and to address sociodemographic differences in the hypertension control cascade.^7,8,9,10^

Our analysis applied the 2017 ACC/AHA guidelines for hypertension.^19^ Prior guidelines from JNC and other organizations (notably, the American Academy of Family Physicians) define hypertension as SBP greater than 140 mm Hg and DBP greater than 90 mm Hg.^27^ Consequently, adults in our study classified as having uncontrolled hypertension according to the 2017 ACC/AHA definition may have met hypertension control criteria using earlier or different guidelines. A previous study^28^ documented increased prevalence of hypertension and of antihypertensive medication recommendations using the 2017 ACC/AHA guideline. Additionally, our results may reflect the slow adoption of the updated guidelines.

Among adults aged 18 to 44 years, the high prevalence and lack of awareness of uncontrolled hypertension is concerning given the importance of early cardiovascular health in preventing negative CVD outcomes later in life.^29^ For females in this age group, uncontrolled hypertension during pregnancy increases the mother’s lifetime risk of CVD and is a leading cause of pregnancy-related death and pregnancy complications.^30,31^ In 2020, hypertensive disorders of pregnancy was the sixth most frequent underlying cause of pregnancy-related death in the US.^32^ Additionally, children born to mothers with uncontrolled hypertension have a greater risk of future adverse health outcomes, including hypertension and CVD.^31^

A prior study^33^ found that hypertension affects approximately 1 in 8 adults aged 20 to 40 years. In our study, the lack of hypertension awareness, and subsequent lack of control among younger adults may reflect this group’s more limited engagement with the health care system compared with older adults.^34^ Even those who are engaged with the health care system are less likely than older adults to be aware of their hypertension status and to subsequently receive and continue treatment for hypertension.^15,35^ Furthermore, studies have demonstrated a lack of persistence in blood pressure lowering among young people following the initial intervention. Additionally, certain life events in young people, such as pregnancy, may require tailored advice from health care professionals on the management of blood pressure.^15,33,35^ Effective management strategies and efforts are needed to increase hypertension awareness among young adults, especially young females. Examples may include improving patient engagement through shared decision-making and assisting patients with obtaining validated self-measured blood pressure monitors.^36^

Our study also revealed a lack of awareness among individuals already engaged with the health care system. More than one-half of adults with uncontrolled hypertension (57.8 million people) remained unaware of their hypertension status, despite nearly 70% reporting 2 or more health care clinician visits within the past year. Previous studies^37^ have documented that poor medication adherence and clinical time pressures, therapeutic inertia, and clinical workloads are barriers to hypertension diagnosis and control. Additionally, despite engagement with the health care system, we found that 70% of adults with uncontrolled hypertension who were aware of their condition reported taking antihypertensive medication. While antihypertensive medications are effective in reducing BP and preventing CVD across demographic groups,^38^ our results support existing evidence that a prescription alone does not guarantee improved hypertension control at the individual or population level. Efforts are needed to improve hypertension awareness and ensure effective control among those prescribed antihypertensive medications.

Evidence-based clinical and community-based efforts can improve outcomes across the hypertension control cascade. Clinical initiatives may include training and evaluation of accurate BP measurement using evidence-based hypertension guidelines, such as the American Medical Association Hypertension Treatment Algorithm.^39^ These guidelines can improve hypertension control through medication treatment intensification, fixed dose combination therapy, nonadherence assessment, and frequent follow-up. Comprehensive process improvements, as outlined in the US Surgeon General’s Call to Action to Control Hypertension,^40^ and the Million Hearts Hypertension and Hypertension in Pregnancy Change Packages^41^ can further support these strategies.

Within the context of these established strategies, future reports and surveillance metrics may support their implementation across the hypertension control cascade. Possible metrics could include increasing health care visits for patients unaware of their hypertension status or with no visits in the past year, enhancing adherence to recommended BP medications, improving medication adherence rates, and increasing clinician adherence to the 2017 AHA/ACC guidelines. A 2023 AHA scientific statement^42^ addressing approaches to improving hypertension control, as well as specific strategies for priority populations, may guide strategies to achieve blood pressure control.^43^ Future research may explore engaging individuals with uncontrolled hypertension, particularly younger adults aged 18 to 44 years, individuals of reproductive age, and those who seldom visit health care clinicians. Enhancing clinical and patient awareness may be key for improving these cascade measures.

### Limitations

Our study has several limitations. First, our findings are not generalizable to individuals who are institutionalized or to military personnel. Second, this study relied on self-reported antihypertensive medication use. Third, NHANES combines several race groups into non-Hispanic other, limiting interpretation and action within this groups. Third, our definition of hypertension is based on BP measurements taken during a single NHANES encounter, but 2017 ACC/AHA guidelines recommend diagnosing hypertension using multiple BP readings from separate occasions.

## Conclusions

This cross-sectional study found a concerning gap in hypertension awareness among adults in the US with uncontrolled hypertension aged 18 to 44 years and those with more than 1 physician visit in the past year. Notably, most adults with uncontrolled hypertension reported using antihypertensive medications. These findings underscore the need for efforts to improve outcomes across levels of the hypertension control cascade.

## Notes

Supplement 1. **eTable.** Age-Standardized Hypertension Cascade Prevalence Estimates Among Adults Aged 18 Years or Older in the US with Hypertension by Sociodemographic and Health Characteristics, January 2017-March 2020 **eFigure 1. **Hypertension Control Cascade Population Estimates Among Adults Aged 18 Years or Older in the US with Uncontrolled Hypertension by Age and Race and Ethnicity and Stratified by Sex, January 2017-March 2020 **eFigure 2. **Hypertension Control Cascade Population Estimates Among Adults Aged 18 Years or Older in the US with Uncontrolled Hypertension by Select Risk Factors and Sociodemographic Variables, January 2017-March 2020

Supplement 2.
Data Sharing Statement
