# Supplementary material for: Examining the Hypertension Control Cascade in Adults With Uncontrolled Hypertension in the US
Source: JAMA Netw Open. 2024 Sep 11;7(9):e2431997. doi: 10.1001/jamanetworkopen.2024.31997 (PMC11391330; doi:10.1001/jamanetworkopen.2024.31997)
Supplement: Supplement 1. — eTable. Age-Standardized Hypertension Cascade Prevalence Estimates Among Adults Aged 18 Years or Older in the US with Hypertension by Sociodemographic and Health Characteristics, January 2017-March 2020 eFigure 1. Hypertension Control Cascade Population Estimates Among Adults Aged 18 Years or Older in the US with Uncontrolled Hypertension by Age and Race and Ethnicity and Stratified by Sex, January 2017-March 2020 eFigure 2. Hypertension Control Cascade Population Estimates Among Adults Aged 18 Years or Older in the US with Uncontrolled Hypertension by Select Risk Factors and Sociodemographic Variables, January 2017-March 2020 [file jamanetwopen-e2431997-s001.pdf]

Supplemental Online Content

Richardson LC, Vaughan AS, Wright JS, Coronado F. Examining the hypertension control cascade in adults with uncontrolled hypertension in the US. *JAMA Netw. Open*. 2024;7(9):e2431997. doi:10.1001/jamanetworkopen.2024.31997

eTable. Age-Standardized Hypertension Cascade Prevalence Estimates Among Adults Aged 18 Years or Older in the US with Hypertension by Sociodemographic and Health Characteristics, January 2017-March 2020

eFigure 1. Hypertension Control Cascade Population Estimates Among Adults Aged 18 Years or Older in the US with Uncontrolled Hypertension by Age and Race and Ethnicity and Stratified by Sex, January 2017-March 2020

eFigure 2. Hypertension Control Cascade Population Estimates Among Adults Aged 18 Years or Older in the US with Uncontrolled Hypertension by Select Risk Factors and Sociodemographic Variables, January 2017-March 2020

This supplemental material has been provided by the authors to give readers additional information about their work.

© 2024 Richardson LC et al. *JAMA Network Open.*

eTable. Age-Standardized Hypertension Cascade Prevalence Estimates Among Adults Aged 18 Years or Older in the US with Hypertension by Sociodemographic and Health Characteristics, January 2017-March 2020

	Sample Size (Estimated population, in millions)	% (95% CI)
**Normal BMI**		
Uncontrolled hypertension [a]	611 (17.5)	88.3 (79.9 - 95.4)
Unaware, not recommended treatment [b]	340 (11.3)	64.9 (51.1 - 78.6)
Aware, met criteria for lifestyle modifications [c]	suppressed	suppressed
Aware, met criteria for lifestyle modifications plus medication [c]	suppressed	suppressed
Aware, currently taking BP medication [d]	suppressed	suppressed
**Overweight**		
Uncontrolled hypertension [a]	958 (31.2)	85.4 (80.4 - 90.5)
Unaware, not recommended treatment [b]	454 (19.0)	60.8 (52.1 - 69.5)
Aware, met criteria for lifestyle modifications [c]	suppressed	suppressed
Aware, met criteria for lifestyle modifications plus medication [c]	483 (10.4)	84.7 (72.8 - 96.4)
Aware, currently taking BP medication [d]	406 (7.6)	73.7 (61.6 - 85.8)
**Obese**		
Uncontrolled hypertension [a]	1505 (50.7)	81.0 (76.3 - 85.6)
Unaware, not recommended treatment [b]	615 (26.7)	52.7 (47.3 - 58.1)
Aware, met criteria for lifestyle modifications [c]	51 (4.0)	16.7 (9.9 - 23.6)
Aware, met criteria for lifestyle modifications plus medication [c]	839 (20.0)	83.3 (76.4 - 90.1)
Aware, currently taking BP medication [d]	680 (14.2)	71.0 (61.4 - 80.6)
**Less than high school education**		
Uncontrolled hypertension [a]	612 (12.2)	88.1 (82.8 - 93.3)
Unaware, not recommended treatment [b]	271 (6.9)	56.3 (46.5 - 66.0)
Aware, met criteria for lifestyle modifications [c]	suppressed	suppressed
Aware, met criteria for lifestyle modifications plus medication [c]	335 (5.0)	93.1 (87.8 - 98.5)
Aware, currently taking BP medication [d]	277 (3.4)	68.7 (54.2 - 83.1)
**High school graduate**		
Uncontrolled hypertension [a]	784 (29.5)	82.7 (76.4 - 89.1)
Unaware, not recommended treatment [b]	346 (17.1)	57.9 (48.5 - 67.3)
Aware, met criteria for lifestyle modifications [c]	21 (2.0)	16.3 (6.5 - 26.1)
Aware, met criteria for lifestyle modifications plus medication [c]	417 (10.4)	83.7 (73.9 - 93.5)
Aware, currently taking BP medication [d]	339 (7.6)	73.3 (59.5 - 87.2)
**Some college**		
Uncontrolled hypertension [a]	1027 (32.0)	83.3 (78.8 - 87.7)
Unaware, not recommended treatment [b]	456 (17.8)	55.5 (49.1 - 61.9)
Aware, met criteria for lifestyle modifications [c]	35 (2.5)	17.5 (8.8 - 26.2)
Aware, met criteria for lifestyle modifications plus medication [c]	536 (11.8)	82.5 (73.8 - 91.2)
Aware, currently taking BP medication [d]	431 (7.7)	65.8 (54.2 - 77.5)
**College graduate**		
Uncontrolled hypertension [a]	681 (26.2)	83.3 (77.7 - 88.9)
Unaware, not recommended treatment [b]	349 (15.9)	60.4 (51.2 - 69.6)
Aware, met criteria for lifestyle modifications [c]	suppressed	suppressed

© 2024 Richardson LC et al. *JAMA Network Open.*

	Sample Size (Estimated population, in millions)	% (95% CI)
Aware, met criteria for lifestyle modifications plus medication [c]	suppressed	suppressed
Aware, currently taking BP medication [d]	suppressed	suppressed
**FIPR <130%**		
Uncontrolled hypertension [a]	765 (15.7)	85.8 (80.4 - 91.3)
Unaware, not recommended treatment [b]	331 (8.6)	54.6 (44.5 - 64.8)
Aware, met criteria for lifestyle modifications [c]	suppressed	suppressed
Aware, met criteria for lifestyle modifications plus medication [c]	420 (6.6)	92.4 (86.4 - 98.4)
Aware, currently taking BP medication [d]	327 (4.6)	69.8 (59.4 - 80.2)
**FIPR 131-350%**		
Uncontrolled hypertension [a]	1099 (33.5)	86.2 (81.4 - 90.9)
Unaware, not recommended treatment [b]	493 (18.6)	55.6 (48.1 - 63.0)
Aware, met criteria for lifestyle modifications [c]	37 (2.7)	18.4 (8.9 - 27.9)
Aware, met criteria for lifestyle modifications plus medication [c]	569 (12.1)	81.6 (72.1 - 91.1)
Aware, currently taking BP medication [d]	470 (8.2)	67.8 (55.5 - 80.2)
**FIPR >350%**		
Uncontrolled hypertension [a]	859 (40.5)	81.7 (77.0 - 86.4)
Unaware, not recommended treatment [b]	431 (24.5)	60.5 (53.2 - 67.7)
Aware, met criteria for lifestyle modifications [c]	27 (3.8)	23.6 (11.7 - 35.6)
Aware, met criteria for lifestyle modifications plus medication [c]	401 (12.2)	76.4 (64.4 - 88.3)
Aware, currently taking BP medication [d]	suppressed	suppressed
**Private health insurance**		
Uncontrolled hypertension [a]	1569 (60.9)	83.1 (79.1 - 87.0)
Unaware, not recommended treatment [b]	748 (35.7)	58.6 (52.8 - 64.4)
Aware, met criteria for lifestyle modifications [c]	53 (5.6)	22.3 (13.9 - 30.6)
Aware, met criteria for lifestyle modifications plus medication [c]	768 (19.6)	77.7 (69.4 - 86.1)
Aware, currently taking BP medication [d]	648 (15.1)	77.0 (66.9 - 87.2)
**Medicare**		
Uncontrolled hypertension [a]	619 (14.4)	77.3 (65.8 - 87.0)
Unaware, not recommended treatment [b]	suppressed	suppressed
Aware, met criteria for lifestyle modifications [c]	suppressed	suppressed
Aware, met criteria for lifestyle modifications plus medication [c]	408 (7.6)	97.8 (94.4 -100.0)
Aware, currently taking BP medication [d]	suppressed	suppressed
**Other health insurance**		
Uncontrolled hypertension [a]	504 (13.4)	84.4 (76.6 - 92.2)
Unaware, not recommended treatment [b]	230 (6.6)	49.2 (36.4 - 62.0)
Aware, met criteria for lifestyle modifications [c]	suppressed	suppressed
Aware, met criteria for lifestyle modifications plus medication [c]	257 (6.1)	89.8 (82.1 - 97.3)
Aware, currently taking BP medication [d]	suppressed	suppressed
**No health insurance**		
Uncontrolled hypertension [a]	421 (10.8)	92.0 (83.9 - 98.5)
Unaware, not recommended treatment [b]	245 (6.4)	58.8 (48.9 - 68.8)
Aware, met criteria for lifestyle modifications [c]	17 (0.7)	16.3 (7.6 - 25.4)

© 2024 Richardson LC et al. *JAMA Network Open.*

	Sample Size (Estimated population, in millions)	% (95% CI)
Aware, met criteria for lifestyle modifications plus medication [c]	159 (3.7)	83.7 (74.6 - 92.4)
Aware, currently taking BP medication [d]	92 (1.9)	50.4 (34.9 - 64.8)
**No healthcare visits**		
Uncontrolled hypertension [a]	430 (13.0)	97.1 (93.9 - 99.9)
Unaware, not recommended treatment [b]	318 (9.9)	75.7 (64.4 - 87.0)
Aware, met criteria for lifestyle modifications [c]	18 (0.7)	23.6 (8.8 - 38.6)
Aware, met criteria for lifestyle modifications plus medication [c]	94 (2.4)	76.4 (61.4 - 91.2)
Aware, currently taking BP medication [d]	suppressed	suppressed
**1 healthcare visit**		
Uncontrolled hypertension [a]	511 (16.1)	90.3 (85.3 - 94.9)
Unaware, not recommended treatment [b]	308 (10.1)	62.7 (51.7 - 73.7)
Aware, met criteria for lifestyle modifications [c]	suppressed	suppressed
Aware, met criteria for lifestyle modifications plus medication [c]	suppressed	suppressed
Aware, currently taking BP medication [d]	suppressed	suppressed
**≥ 2 healthcare visits**		
Uncontrolled hypertension [a]	2181 (70.6)	79.7 (75.3 - 84.1)
Unaware, not recommended treatment [b]	812 (36.6)	51.8 (46.5 - 57.1)
Aware, met criteria for lifestyle modifications [c]	50 (5.0)	14.7 (8.0 - 21.4)
Aware, met criteria for lifestyle modifications plus medication [c]	1319 (29.0)	85.3 (78.6 - 92.0)
Aware, currently taking BP medication [d]	1147 (23.0)	79.4 (72.8 - 86.0)
**Chronic Kidney Disease**		
Uncontrolled hypertension [a]	806 (22.3)	79.6 (72.0 - 87.2)
Unaware, not recommended treatment [b]	229 (9.0)	40.6 (27.4 - 53.8)
Aware, met criteria for lifestyle modifications [c]	0 (0.0)	0.0 (0.0 - 0.0)
Aware, met criteria for lifestyle modifications plus medication [c]	577 (13.2)	100.0 (100.0 -100.0)
Aware, currently taking BP medication [d]	487 (8.2)	62.3 (48.4 - 76.2)
**Diabetes**		
Uncontrolled hypertension [a]	806 (21.9)	76.7 (69.2 - 84.2)
Unaware, not recommended treatment [b]	240 (8.8)	40.2 (26.9 - 53.5)
Aware, met criteria for lifestyle modifications [c]	0 (0.0)	0.0 (0.0 - 0.0)
Aware, met criteria for lifestyle modifications plus medication [c]	566 (13.1)	100.0 (100.0 -100.0)
Aware, currently taking BP medication [d]	503 (9.7)	74.1 (59.1 - 89.1)
**History of ASCVD**		
Uncontrolled hypertension [a]	561 (16.8)	78.0 (66.3 - 88.7)
Unaware, not recommended treatment [b]	suppressed	suppressed
Aware, met criteria for lifestyle modifications [c]	0 (0.0)	0.0 (0.0 - 0.0)
Aware, met criteria for lifestyle modifications plus medication [c]	440 (12.2)	100.0 (100.0 -100.0)
Aware, currently taking BP medication [d]	375 (9.1)	74.9 (60.2 - 89.6)

[a] Among all adults with hypertension

[b] Among adults with uncontrolled hypertension; individuals who were unaware of their hypertension status were considered to not have been recommended treatment.

© 2024 Richardson LC et al. *JAMA Network Open.*

[c] Among adults with uncontrolled hypertension who were aware of their hypertension status.
[d] Among adults with uncontrolled hypertension who were aware of their hypertension status and met the 2017 criteria for lifestyle modifications plus medication.

© 2024 Richardson LC et al. *JAMA Network Open.*

eFigure 1. Hypertension Control Cascade Population Estimates Among Adults Aged 18 Years or Older in the US with Uncontrolled Hypertension by Age and Race and Ethnicity and Stratified by Sex, January 2017-March 2020

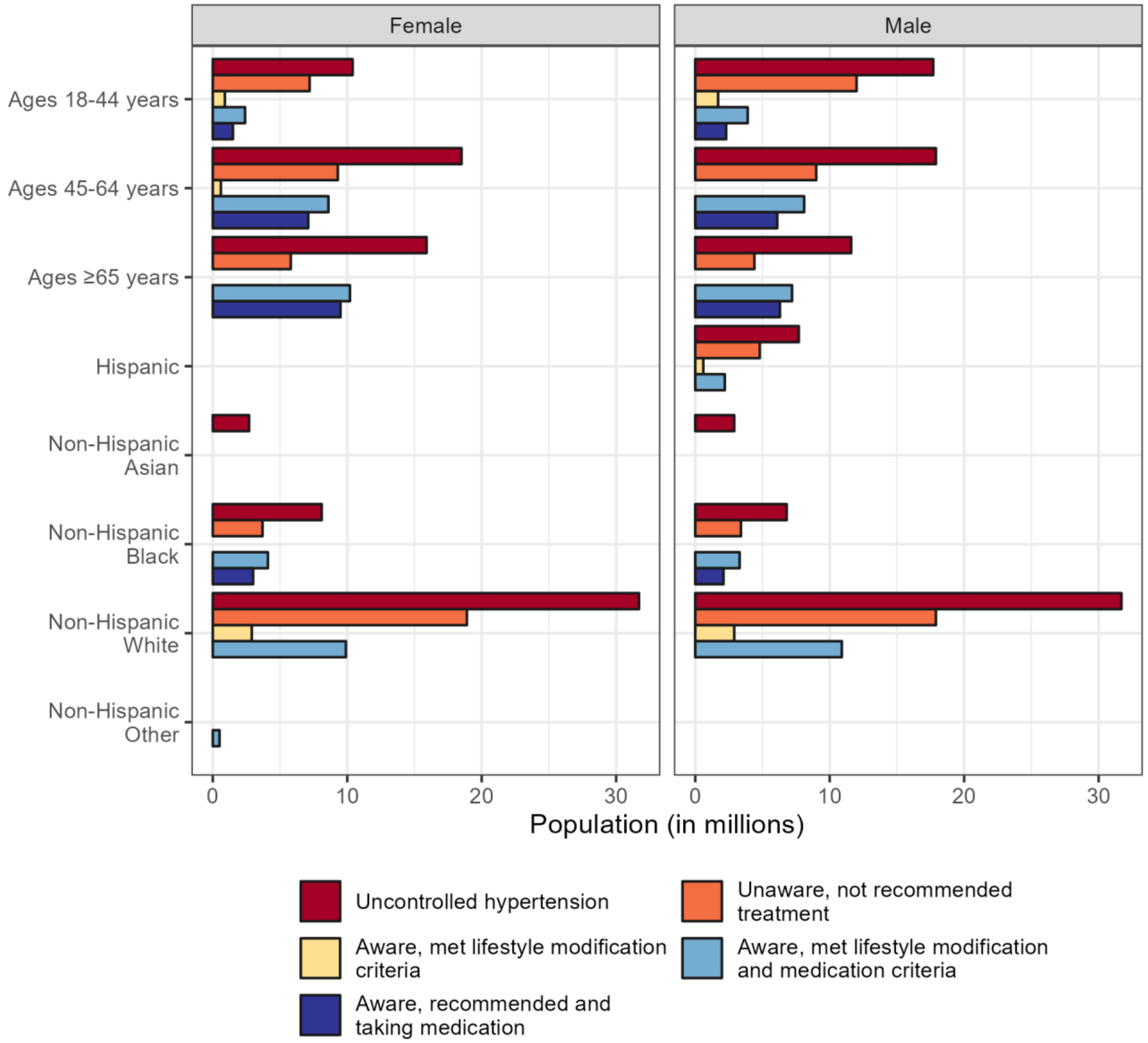

© 2024 Richardson LC et al. *JAMA Network Open.*

eFigure 2. Hypertension Control Cascade Population Estimates Among Adults Aged 18 Years or Older in the US with Uncontrolled Hypertension by Select Risk Factors and Sociodemographic Variables, January 2017-March 2020

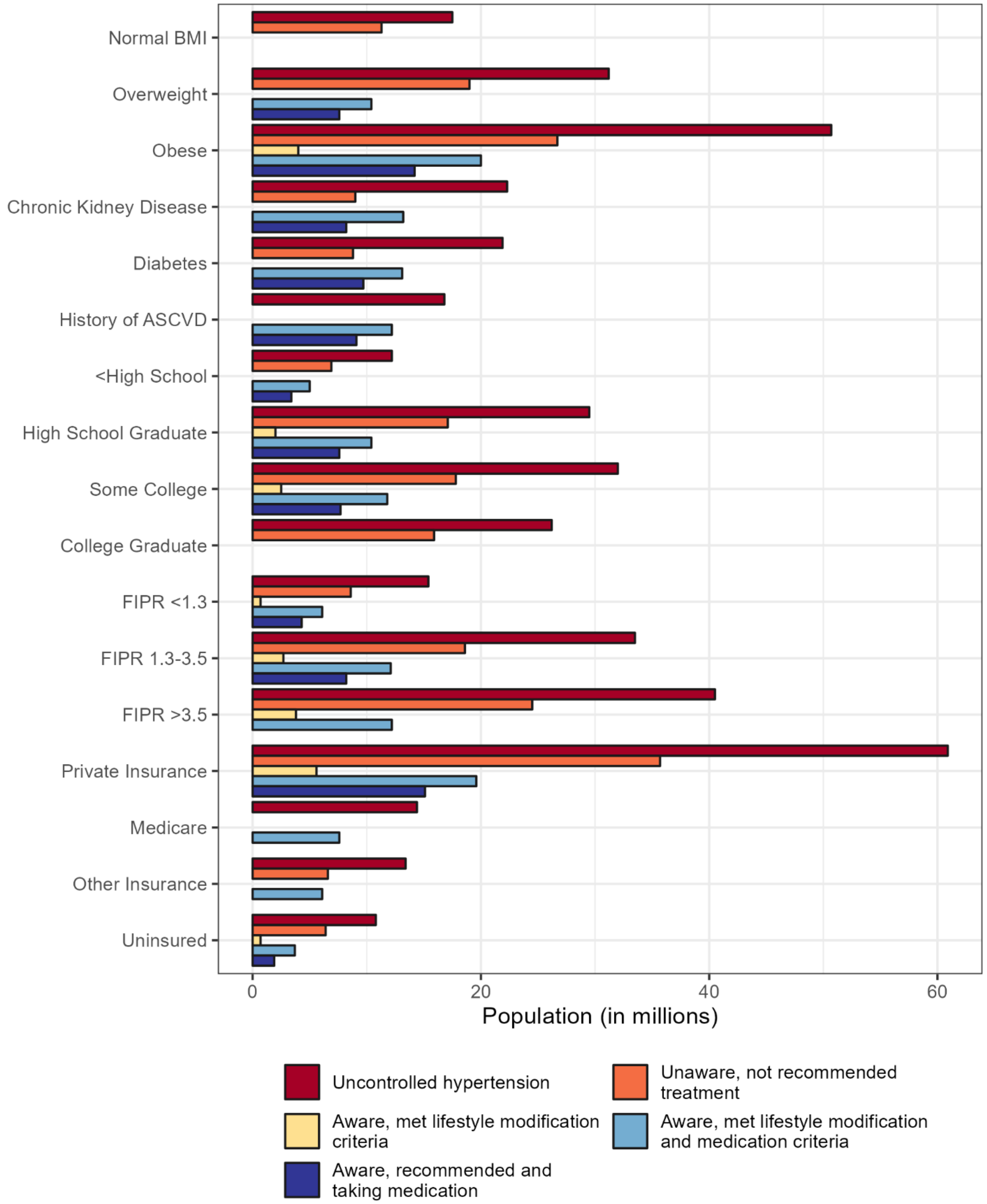

© 2024 Richardson LC et al. *JAMA Network Open.*
