# Supplementary material for: Examining the Hypertension Control Cascade in Adults With Uncontrolled Hypertension in the US
Source: JAMA Netw Open. 2024 Sep 11;7(9):e2431997. doi: 10.1001/jamanetworkopen.2024.31997 (PMC11391330; doi:10.1001/jamanetworkopen.2024.31997)
Supplement: Supplement 2. — Data Sharing Statement [file jamanetwopen-e2431997-s002.pdf]

Data Sharing Statement

Richardson. Examining the Hypertension Control Cascade in Adults With Uncontrolled Hypertension in the US. *JAMA Netw Open*. Published September 11, 2024. doi:10.1001/jamanetworkopen.2024.31997

Data
Data available: Yes
Data types: Deidentified participant data
How to access data: The NHANES data used in our study are publicly available at: https://wwwn.cdc.gov/nchs/nhanes/Search/DataPage.aspx?Component=Examination&Cycle=2017-2020
When available: With publication

Supporting Documents
Document types: None

Additional Information
Who can access the data: The NHANES data used in our study are publicly available at: https://wwwn.cdc.gov/nchs/nhanes/Search/DataPage.aspx?Component=Examination&Cycle=2017-2020
Types of analyses: The NHANES data used in our study are publicly available at: https://wwwn.cdc.gov/nchs/nhanes/Search/DataPage.aspx?Component=Examination&Cycle=2017-2020
Mechanisms of data availability: The NHANES data used in our study are publicly available at: https://wwwn.cdc.gov/nchs/nhanes/Search/DataPage.aspx?Component=Examination&Cycle=2017-2020
